# Graphene platelets from shungite rock modulate electropolymerization and charge storage mechanisms of soft-template synthetized polypyrrole-based nanocomposites

**DOI:** 10.1038/s41598-018-35415-2

**Published:** 2018-11-19

**Authors:** Sara Politi, Rocco Carcione, Emanuela Tamburri, Roberto Matassa, Teresa Lavecchia, Mariglen Angjellari, Maria Letizia Terranova

**Affiliations:** 1 Dip. to di Scienze e Tecnologie Chimiche, Università degli Studi di Roma “Tor Vergata”, Via della Ricerca Scientifica, 00133 Rome, Italy; 2 Department of Anatomical, Histological, Forensic and Orthopaedic Sciences, Section of Human Anatomy, Sapienza University of Rome, Via A, Borelli 50, Rome, Italy

## Abstract

We report here on soft-template electropolymerizations of polypyrrole (Ppy)-based nanocomposites triggered by graphene platelets (GP) from shungite (SH) rocks. A properly designed procedure for an efficient extraction of graphene platelets from SH powders is established to produce remarkable graphene materials in a low oxidation state and with a high electrical conductivity (1490 S cm^−1^). By using positively and negatively charged templating surfactants the role played by the graphene units on the electropolymerization reactions is pointed out by SEM, EDX, TEM, SAED, XPS and Raman spectroscopy. The morphological/structural characterizations highlight that GP from SH have a surface chemistry suitable for selective and mutual interactions with the growing Ppy chains. CV and galvanostatic charge/discharge measurements evidence that GP improve the transport of both electrons and ions within the bulk material by means of a synergistic action with the polymer phase. This cooperative behavior induces an enhancement of the specific capacitance up to 250 F g^−1^ at 2 A g^−1^. The Ppy-GP materials produced following the settled protocols result to be appropriate for fabricating multifunctional charge transport and storage electroactive systems.

## Introduction

The manufacturing of organic-inorganic hybrids and nanocomposites is a key cross-cutting technology for creating high-performance and high-functionality materials broadly applicable and pervasive in multiple industries and markets. In particular, when the goal is to fabricate a multicomponent system with synergistic functionalities among the constituents, the choice of the appropriate precursors and the methodology for the preparation of the final material represent a critical step^1^. This challenging task is presently accomplished thanks to coupling organic polymers with selectively chosen inorganic nanosized fillers, and to engineering hybrid nanomaterials or nanostructured composite systems^2^. The use as a filler of basic inorganic entities, such as nanoclays, silicates, oxides and carbon nanostructures, has opened the way for advanced applications in energy generation and storage, electronics, sensors, catalysis and medical devices^3^.

In this context carbon systems have always occupied a prominent position in the inorganic materials field. The first applications of carbons to solve technical problems date back to the IV millennium B.C. and, along the millennia, natural carbon forms have been more and more employed up to recent times, in which we are assisting to an upsurge of artificially fabricated C structures. Each of the natural C allotropes can be now replicated by man-made structures, and presently the large family of artificial carbon materials spans a continuum from sp^1^ to sp^2^ and sp^3^ configurations at multiple length scales, down to the reduced dimensions of the nano-world. In this frame, nowadays materials scientists are skilled at designing and constructing unconventional carbons with precise nanometer-level forms and organizations.

Among the artificial sp^2^-carbons, graphene and graphene–like nanostructures have become ones of the most studied materials to date. They encompass a wide variety of systems that are classified on the basis of average lateral dimension, number of layers, or C/O ratio, which allow them to be ideal building blocks of more sophisticated complex systems, as paramount additives in multiphase materials or biomaterials in nanomedicine and bio-related devices^4^. Commonly, the mass-production of these systems is accomplished from graphite by adopting physical methods (mechanical exfoliation by ultrasound or thermal treatments) or chemical approaches (intercalation using organic or inorganic substances)^5–10^. The structures obtained from the first methods are generally high quality graphene sheets with a good degree of crystallinity, limited defect density, and high conductivity. However, their yield is usually quite low. By chemical treatments, the yield is higher but the graphene sheets contain a number of defects in the π−conjugated network, that induce a lowering of the electrical conductivity^11,12^.

In the continuous race to develop alternative methodologies for the large-scale production of what has been hailed as the future’s 2-D miracle material, we would like to emphasize how some “old” components of the carbon family may be viewed as unexpected sources of graphene nanostructures. In this context high regard must be given to shungite (SH)^13^, a natural carbon-based mineraloid originating from an area of the Karelia region (Russia), that has for centuries been empirically known and successfully used for its practical properties. Typically, shungite rocks are classified according to their carbon content that is closely connected to the geological site they comes from. In particular, the deposits of shungite having the more economic significance can provide boulders for which the carbon content is 98–100%_wt_^14^.

The peculiar organization at the nanoscale of the carbon phase constituting the SH rocks has been highlighted by several morphological and structural studies, that have evidenced the presence of a multilevel fractal structure of sp^2^-C entities formed by graphene-like arrangements^15–25^. The consequent revelation that the fundamental carbon component forming the microstructure of this raw material can also be given by nano-sized agglomerates of graphene-like systems^15,17–25^, is feeding an increasing scientific and technological interest towards this fascinating natural carbon allotrope. In fact, the SH-derived carbon is nowadays object of many exciting speculations, and high attention is paid to identifying the technological areas in which its properties can be exploited to the best^16,26–33^.

On the other hands, among the several classes of polymeric materials, conducting polymers occupy a key role in developing devices with advanced functionalities^34^. Owing to the electroactivity and the intrinsic electronic conduction, conducting polymers have been proposed and are presently utilized in several fields of application ranging from organic electronics, to biomedical platforms, and energy storage and conversion devices. In order to improve features and functionalities of these systems, a reliable solution was found in the integration with nanocarbons for producing nanocomposite materials^35–38^.

In particular, interesting findings have pointed out the effect produced by C nanostructures during the polymerization reaction. In the case of nanodiamond, it was demonstrated how the nanosized dimensions and the surface properties of this material were able to modulate the 3-D organization of polymer chains and the functional properties of the final material^39–45^. In this perspective, we felt worthwhile to investigate whether the nanostructured carbon materials composing SH could participate in the polymerization process by guiding the arrangement of polymer backbones.

In order to isolate the carbon phase from shungite, we tested several chemical purification treatments. The graphene platelets (GP) successfully extracted from the rock were submitted to an in-depth surface and composition/structural characterization, and were coupled with pyrrole monomers during *in situ* soft-template electropolymerization reactions. Both positively and negatively charged surfactants were used as monomer dispersing agents and polypyrrole polymer dopants. The nature of the interfacial interactions between GP and the various micellar templates were assessed thus permitting to rationalize the properties exhibited by the final hybrid materials. In particular, a thorough investigation of the structure and morphology of the nanocomposites produced under different experimental conditions, as well as of the modifications induced by the GP filler to the functional electroactivity of the polymer matrix was performed in order to reach a comprehensive knowledge of these novel hybrid systems.

## Experimental

### Materials and Methods

The SH used for the present experiments was a powder of type-III shungite rock, gently provided by the Geology Institute of the Karelian Research Centre of Russian Academy of Sciences. It comes from deposits of diapirs which are organosiliceous rocks with a variable amount of the main C (20–55%_wt_) and SiO_2_ (35–75%_wt_) components. The settling of efficient purification procedures able to eliminate the silicates and other species present in the pristine SH rock and to obtain a network of pure carbon phase, is undoubtedly a fundamental step preliminary to any other experimental activity. Based on literature considerations about the use of HF or NaOH to eliminate silicates^46^, of HNO_3_ or HCl to remove metals, oxides and amorphous carbon^47^, new purification procedures were settled by combining some of the proposed reactants. The treatments performed on the pristine SH powder are the following:

**A** - overnight dissolution in a 23 M HF aqueous solution, followed by 16 M HNO_3_ and 12 M HCl aqueous solution treatments for 5 hours and overnight, respectively;

**B** - overnight dissolution in a 23 M HF aqueous solution, followed by an overnight treatment in 12 M HCl aqueous solution;

**C** - preparation of a fused NaOH/SH phase, kept in a furnace at 520 °C for 3 h, followed by a 16 M HNO_3_ and 12 M HCl aqueous solution treatments for 5 hours and overnight, respectively;

**D** - dissolution in a 1:1 mixture of 23 M HF and 16 M HNO_3_ aqueous solution for 10 h, followed by an overnight treatment in a 12 M HCl aqueous solution.

All the treatments were carried out under agitation by magnetic stirring. At the end of each treatment, the samples were repeatedly washed and centrifuged up to reach a pH = 6. The final powders obtained after drying were collected and characterized. From here on the obtained samples will be referred to A, B, C, D on the basis of the treatment to which they were subjected.

The growth of polypyrrole polymers was carried out starting from 100 mM pyrrole (Aldrich) aqueous solutions. 5 mM sodium dodecyl sulfate (SDS) and 1 mM cetyltrimethylammonium bromide (CTAB) were alternately used for the polymerization reactions. Different amounts of purified SH were used for nanocomposite preparations.

Syntheses of polymer and composite films were performed by chronoamperometry by means of a Palm Sense electrochemical work-station using 3 mm Pt disks as working electrodes, an Ag/AgCl as reference and a Pt wire as counter electrode. Before the electrochemical processes, the Pt disks surfaces were repeatedly polished with 0.5 mm alumina powders, and then washed with distilled water.

The morphology of the samples was analyzed by a FIB-SEM Cross Beam Workstation ZEISS Auriga scanning electron microscope. The apparatus was equipped with a detector for Energy Dispersive X-Ray Spectroscopy (EDX) for elemental analysis. TEM images and Electron Diffraction Patterns (EDPs) were captured by using a FEI-Titan transmission electron microscope operating at 300 keV.

The molecular structure was studied by Raman microspectroscopy using a XploRA ONE^TM^ Raman Microscope (Horiba Jobin Yvon). The instrument setup was provided with two excitation energies (532 and 785 nm), and the Raman-scattered light was detected by an air-cooled scientific CCD camera. The SH-based samples were analyzed under the 532 nm laser light by using a 25% laser power and a diffraction grating of 1200 gr/mm. In order to avoid photoluminescence phenomena, the polymer and nanocomposites samples were analyzed under the 785 laser light by a using 0.1% laser power and a diffraction grating of 2400 gr/mm. All the spectra were fitted using curves with a Lorentzian peak shape.

XPS analysis was performed using a KRATOS Axis Ultra DLD apparatus. An incident monochromated X-ray beam from the Al target (15 kV, 10 mA) was focused on the sample surface. The angle between the X-ray emitter and the photoelectron detector direction was set at 0°. The electron energy analyzer was operated with a pass energy of 20 eV enabling to obtain high resolution spectra. A step size of 0.05 eV was employed.

Electrical conductivity was tested by a transmission current–voltage analysis performed using a two-point probe with two metallic contacts placed onto the samples surface.

The electrochemical behavior of both pure polymers and nanocomposite layers was evaluated by performing cyclic voltammetry and galvanostatic charge/discharge measurements in a standard one-compartment three electrodes cell by means of the Palm Sense work-station. An Ag/AgCl electrode and a Pt coil were respectively used as reference and counter electrode.

## Results and Discussion

### Characterization of the carbon phase extracted from SH

**Figure 1:**
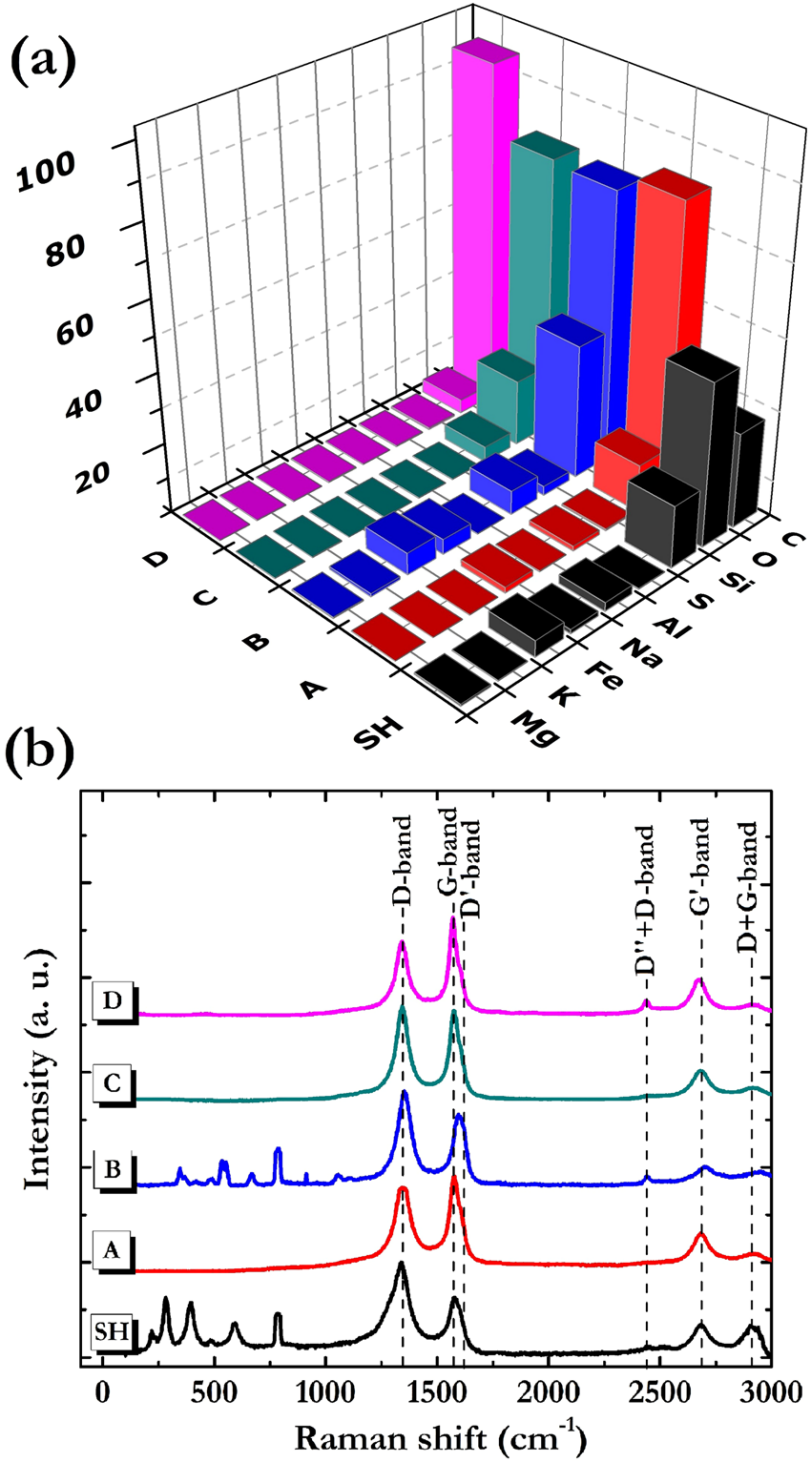
(**a**) Histogram showing the elemental composition and (**b**) Raman spectra of pristine SH powder (black) and of samples treated following the protocols: A (red), B (blue), C (green) and D (magenta).

The effects of purification treatments carried out on the SH powder were assessed by analyzing the elemental composition and the structure of all the produced samples (Fig. 1).

The data summarized in the histogram of Fig. 1a were obtained by averaging several EDX measurements performed on each sample. The analysis shows that carbon, silicon and oxygen are the main components of the pristine rock, whereas S, Al, Na, Fe, Mg and K elements were detected only in traces. Observing the relative elemental concentration of the various purified samples, we note that the most efficient processing in removing silicates and metal oxides species is the D one. Such a sample is given by a 96%_wt_ carbon content, that is equivalent to a 85% yield carbon extraction. Conversely, the other protocols result not so effective to produce high amount of pure carbon.

The structural characterizations were performed by Raman spectroscopy using the 532 nm laser source. In Fig. 1b are shown the Raman spectra of SH, A, B, C, and D samples. In the spectral region below 1000 cm^−1^ the signals related to silicates and metal oxides are clearly observable in the SH spectrum. It is evident that the treatment B is the less efficient in the purification action, since the Raman spectrum of the related sample still shows signals attributable to such not-carbonaceous components.

**Figure 2:**
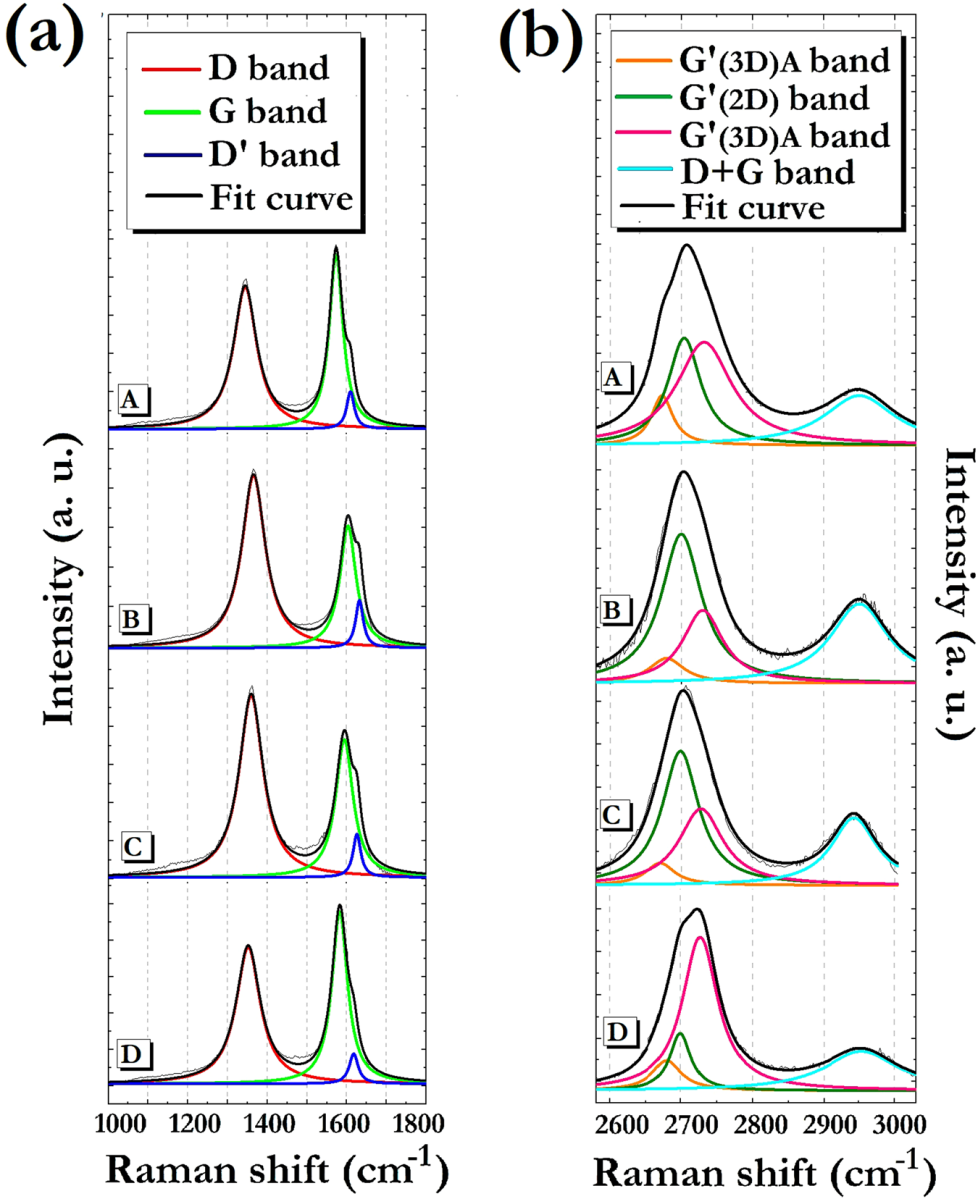
From top to down, deconvolved (**a**) first-order and (**b**) second-order Raman spectra of A, B, C and D samples.

More specific information is achieved by analyzing the spectral zones correlated to carbon materials vibrations. In general, Raman spectra of sp^2^ C systems are characterized by two distinctive spectral regions. The first is located in the 1000–1800 cm^−1^ range and encompasses the first order scattering given by D, G and D′ bands. The G-band occurring at ∼1580 cm^−1^ is produced by the stretching of C-C bonds strongly coupled in hexagonal sheets^48^; the D-band at ∼1350 cm^−1^ is caused by disorder in the sp^2^-hybridized carbon systems^49^, while D′-band found at about 1620 cm^−1^ is associated to defects induced by chemical functionalization^50,51^ or to purely structural factors^52^. The other peculiar spectral range extends from 2550 to 3100 cm^−1^ where are active the second order phonon modes occurring as D″ + D, G′ and D + G bands. The D″ + D signal at ∼2460 cm^−1^ is related to structural defects^53^; the G′ band at about 2700 cm^−1^ corresponds to the overtone of the D band, while the D + G band at about 2950 cm^−1^ is associated to a combination mode induced by disorder effects^54–56^. In the collected spectra we can observe that all the samples exhibit all the described signals at a different extent. Therefore, for a more accurate study of the effects induced by the purification processes, a deconvolution of both spectral regions was performed (Fig. 2).

The analysis of the first-order signals allows for determining the in-plane crystallite mean size of the polycrystalline graphene-based systems, that are the nanostructured materials typically obtained by mass production methodologies. Such an in-plane crystalline dimension is indicated by *La* parameter, that is related to the ratio between the integrated intensities of the disorder-induced D band and the G band (*ID*/*IG*). For *La* evaluation, various empirical equations, that take into account the laser wavelength excitation, have been so far proposed^57–61^. Moreover, a relationship between the FWHM of the G band (FWHM(G)) and the mean diameter of the graphite crystallites has been also found^62^.

In this study *La* was calculated using both the following equations^61,62^:

1 $$ID/IG=12.26/La$$

2 $$FWHM(G)=14+430/La$$

In Table 1 are reported the *La* values calculated by means of the two different formulas.

**Table 1:** *ID*/*IG* ratio, FWHM(G) and *La* values calculated by means of eqs (1) and (2) for the various samples.

Sample	*ID*/*IG*	FWHM (G) (cm^−1^)	*La* from eq. 1 (nm)	*La* from eq. 2 (nm)
A	0.90	47	14	14
B	1.40	54	10	11
C	1.16	62	11	12
D	0.84	43	15	15

It can be observed that for A and D samples the *La* values deriving from eq. (1) coincide with those derived from eq. (2), thus giving a mean graphite crystallite size in the 14–15 nm range. Regarding the B and C samples, the *La* values calculated by eq. (1) are slightly lower than those calculated by eq. (2), and range between 10 and 12 nm. Irrespective of the approach used, however it is clear how shungite can be considered as a source of nanostructured graphitic systems.

Additional information can be obtained by the study of the second order Raman scattering, that can provide an estimation of the out-of-plane crystallite mean size. In particular, the G′ band, an overtone of the D band, is very sensitive to the stacking of graphene sheets along the c axis^63–66^. This means that the analysis of the G′ features permits to discriminate a graphitic system characterized by a regular AB stacking, from a turbostratic graphite possessing variable amounts of stacking faults, or even a complete lack of AB interlayer alignment. It may be useful to recall that such a “disordered” graphite, typically considered as a 2-D system, shows an intense first order D peak^67^ and exhibits a G′ peak (named as G′_(2D)_) up-shifted of about 20 cm^−1^ with respect to that of single-layer graphene. Moreover it is characterized by a FWHM that almost doubles the one of a single graphite sheet^63^. Nevertheless, we should emphasize that in the presence of a certain number of aligned graphene layers (major of 5-6), the shape of the G′ band anyway tends to that of bulk graphite, assimilable to a 3-D structure, and is fitted by the G′_(3D)A_ and G′_(3D)B_ Lorentzian lineshape functions^63–66^.

In the present study we found that the nanostructured graphitic systems coming from SH are characterized by variable amounts of 2-D and 3-D arrangements since all the samples produce a G′ band well-described by three Lorentzian peaks. Moreover, the relative values of integrated intensity of such peaks follow a certain trend. In particular, going from B to C, A and D samples, the IG′_(3D)A_ and IG′_(3D)B_ integrated intensities of the G′_(3D)A_ and G′_(3D)B_ peaks are found to rise at the expense of the integrated intensity IG′_(2D)_ of the G′_(2D)_ peak. This result can be explained by an increase in the stacking order of graphene layers along c-direction.

In order to quantify the decrease of the turbostratic graphite component accomplished using the various purification treatments, the percentage of the disordered graphite (%_(2D)_) was calculated by referring to the integrated intensity values of all the G′ components. The resulting relative amounts are: 34% for A, 60% for B, 55% for C, and 22% for D. These values clearly indicate that the D sample is indubitably the system constituted by the smaller quantity of turbostratic graphite. As a consequence this suggests that the D treatment is the most effective not only in removing the not-carbonaceous impurities present in the starting material, but also in isolating the crystalline sp^2^-C phase present in the SH powder.

**Figure 3:**
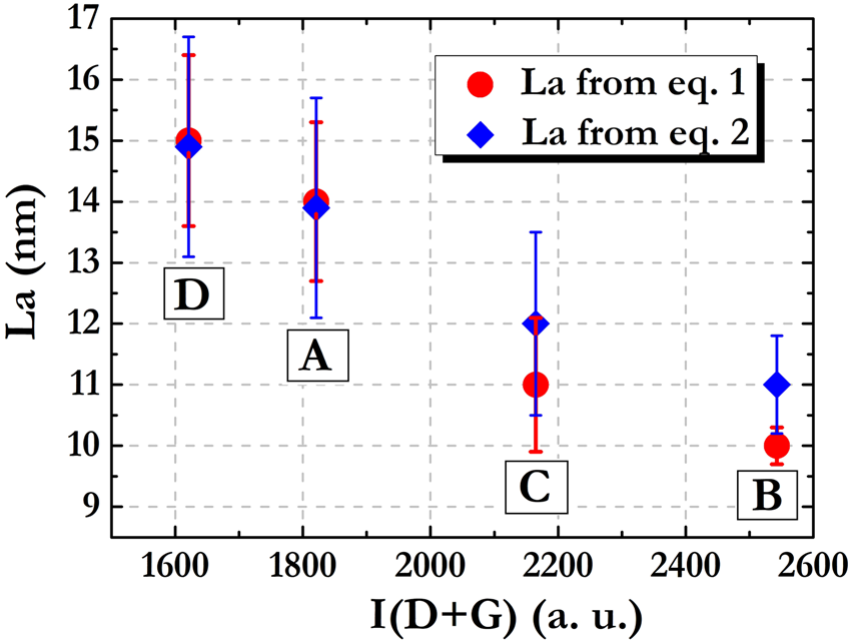
*La* values as a function of the (D + G) band integrated intensity for the various samples. Red: *La* values from eq. (1); Blue: *La* values from eq. (2).

Second-order Raman scattering can also be put in relation with the mean crystallite size of graphitic domains. Several authors observed, in fact, that the intensity of the D + G band at about 2945 cm^−1^ increases by decreasing *La* value^68,69^. In order to check if such a dependence holds for the samples produced by SH purification, the *La* values calculated for the A, B, C, and D powders using both eqs (1) and (2), have been plotted versus the integrated intensity of the D + G band (Fig. 3).

Indeed, B and C samples, which on average give the lowest values for *La*, are characterized by the highest intensity for the D + G band. Samples A and D, for which the largest size of graphite crystallites was obtained, are actually characterized by the less intense D + G band. The agreement between the results given by the analyses of the first and second order Raman signals makes us confident about the employed characterization methodology, and enables to delineate the structural features of the examined materials with a high accuracy. We can reasonably asses that the A and D purification treatments are able to extract the graphitic phase from the raw material, while the B and C protocols are not particularly efficient in isolating the regularly stacked graphene layers. In addition, even if Raman analysis of A, B and C samples did not evidence specific signals attributable to not-carbonaceous species, the EDX measurements revealed that impurities were still present after the A, B and C treatments. In the case of D sample, conversely, a rather complete removal of silicates and metal species was achieved. In conclusion, it can be stated that the chemical procedures constituting D protocol are the most efficient in obtaining materials with an ordered multilayer graphene structure associable to that typically found for graphene nanoplatelets with a 1 to 15 nanometers ranging thick.

### Characterization of graphene platelets

**Figure 4:**
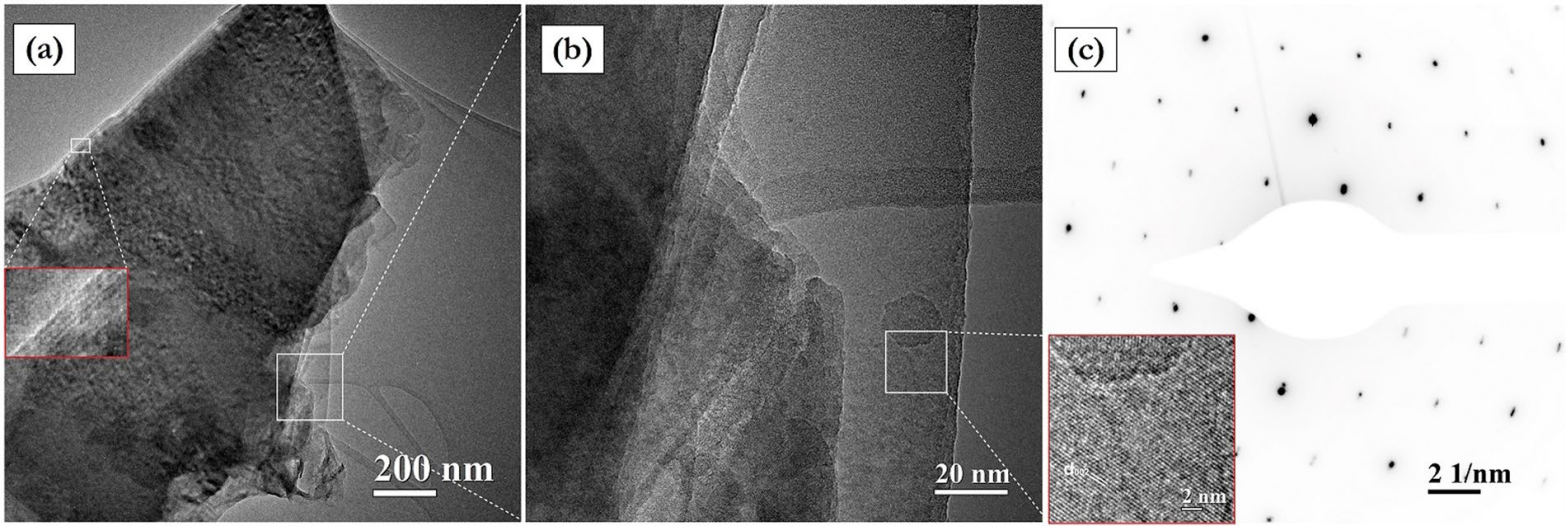
SH purified by means of D treatment. (**a**) BF-TEM image of a platelet. The red squared insert shows a high magnification evidencing a regular stacking of graphene layers. (**b**) BF-TEM high magnification image showing regions with a different number of stacked graphene layers. The red squared insert evidences the graphene crystalline lattice. (**c**) Electron diffraction pattern showing the typical P63/mmc hexagonal crystalline symmetry.

Morphology and structure of D sample were studied in a greater detail by means of bright-field (BF) transmission electron microscopy (TEM)^70^, that emphasized the actual presence of platelets with a micrometric surface and a ten of nanometers thickness. In particular, the red squared insert in the BF-TEM image of Fig. 4a confirms that such platelets are given by an ordered stacking of a few graphene layers.

Additional observations were performed to highlight the nanometric features of such system. BF-TEM image of Fig. 4b shows how the platelet is characterized by regions with a different intensity of contrast. In particular, the latter varies from a high level on the left side to reach a low level on the right one depending on the number of stacked graphene layers, the crystalline lattice of which is, anyway, clearly evident on the entire area^71^. Accurate measurements of the lattice fringes showed a preferential inter-planar spacing of *d*_002_ = 0.335 nm belonging to the P63/mmc hexagonal symmetry of graphite, as shown in the red squared insert. This reference was exploited for estimating the nanometric dimension of the crystal lattices with a reasonable accuracy, as a subnanometer calibration of HR-TEM image^72^. A further analysis aimed at definitively evaluate the GP crystalline structure was performed by selected area electron diffraction (SAED) measurements. Figure 4c distinctly shows diffraction spots ordered into a hexagonal pattern which confirms the crystallographic features of stacked graphene layers oriented along the [111] zone axis parallel to the electron beam. Therefore, the morphological and structural analyses performed by coupling HR-TEM and SAED techniques provided a valid confirm of the tendency of the graphene layers to assemble into platelets after the purification process.

**Figure 5:**
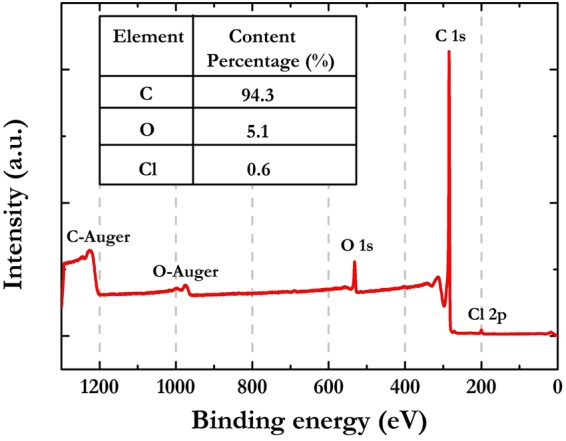
XPS survey spectrum with enclosed GP elemental composition.

The surface chemical composition of GP was analyzed by XPS. In Fig. 5 is reported a typical XPS survey spectrum taken from the sample.

As one can observe, C, O, Cl are the only elements present on the GP surface. By extracting the core lines from the survey it was possible to obtain a quantitative evaluation, as shown in the insert of Fig. 5. The data obtained from XPS analysis are reasonably consistent with those obtained by EDX measurements. The negligible amount of chlorine found by XPS can be due to some HCl remaining on the sample surface after the purification treatment. The high C/O ratio (about 20) points out that the obtained GP are formed by graphene layers with a few oxygen-containing groups. This last finding is rather remarkable because the electrical behavior of graphene-like materials is strictly related to the oxygen content, which is associated to sp^2^ network defects and responsible for the conductivity decrease of the various graphene oxide (GO) forms. The reduction treatments typically performed to produce rGO systems and restore the conductivity of the graphitic domains, present in fact several drawbacks^73^. Therefore, the settling of procedures able to generate graphene platelets characterized by a low amount of oxygenated functionalities can be considered a particularly significant task.

The conductivity of the purified graphene phase obtained from SH was achieved from the GP powder pelletized by means of a hydraulic press. It is worthy to emphasize that the average value of 1490 ± 100 S cm^−1^ found for GP from SH matches that of GP obtained by graphite using physical methods, while is definitely higher than the values typically exhibited by rGO materials^73–75^.

### Preparation of PpyGP nanocomposites

The GP deriving from the D treatment were utilized as a filler for the production of polymer-based nanocomposites. An *in situ* synthesis was adopted for the preparation of the nanocomposites, by means of a monomer electropolymerization in aqueous media where GP were previously dispersed. Two different molar ratios of pyrrole and GP, namely 1:1 and 1:3, were used for the experiments. In Table 2 are summarized the data related to the samples preparation.

**Table 2:** Synthesis parameters and experimental data related to the polymers and nanocomposites preparation.

sample	Ppy:GP molar ratio	Electro-polymerization conditions	Polymerization electrical charge (mC cm^−2^)	Mass (µg)
DSPpy	—	E_0_ = 0.2 V, t_0_ = 10 s; E_1_ = + 0.8 V, t_1_ = 300 s	1050	49
DSPpyGP1	1:1	E_0_ = 0.2 V, t_0_ = 10 s; E_1_ = + 0.8 V, t_1_ = 300 s	1600	78
DSPpyGP3	1:3	E_0_ = 0.2 V, t_0_ = 10 s; E_1_ = + 0.8 V, t_1_ = 300 s	2100	105
CTAPpy	—	E_0_ = 0.2 V, t_0_ = 10 s; E_1_ = + 1.2 V, t_1_ = 300 s	180	10
CTAPpyGP1	1:1	E_0_ = 0.2 V, t_0_ = 10 s; E_1_ = + 1.2 V, t_1_ = 300 s	1100	32
CTAPpyGP3	1:3	E_0_ = 0.2 V, t_0_ = 10 s; E_1_ = + 1.2 V, t_1_ = 300 s	1400	48

Aiming at facilitate the dispersions, two differently charged surfactants were added at their critical micelle concentration. Specifically, the idea was to assess the effects of the two dispersants towards the interaction between pyrrole monomers and GP during the polymerization process. For the purpose, the anionic SDS and the cationic CTAB surfactants were investigated. These systems were exploited as support electrolytes of the electropolymerization processes, and at the same time as doping agents for the polymer matrix. In order to highlight the growth mechanism of the polymer systems, pure polymer samples were also prepared under the same conditions adopted for the composites preparation.

Since the electropolymerization is an oxidative reaction, the use of differently charged surfactants was immediately reflected into the different anodic polarization required for the formation of pyrrole radical monomers. In particular, after double layer instauration at +0.2 V, potentials of +0.8 V and +1.2 V were necessary to apply for pyrrole polymerization in SDS and CTAB solution, respectively. This can be rationalized on the basis of the different interactions between the specific surfactant (SDS or CTAB) and the surface of the positively polarized electrode.

**Figure 6:**
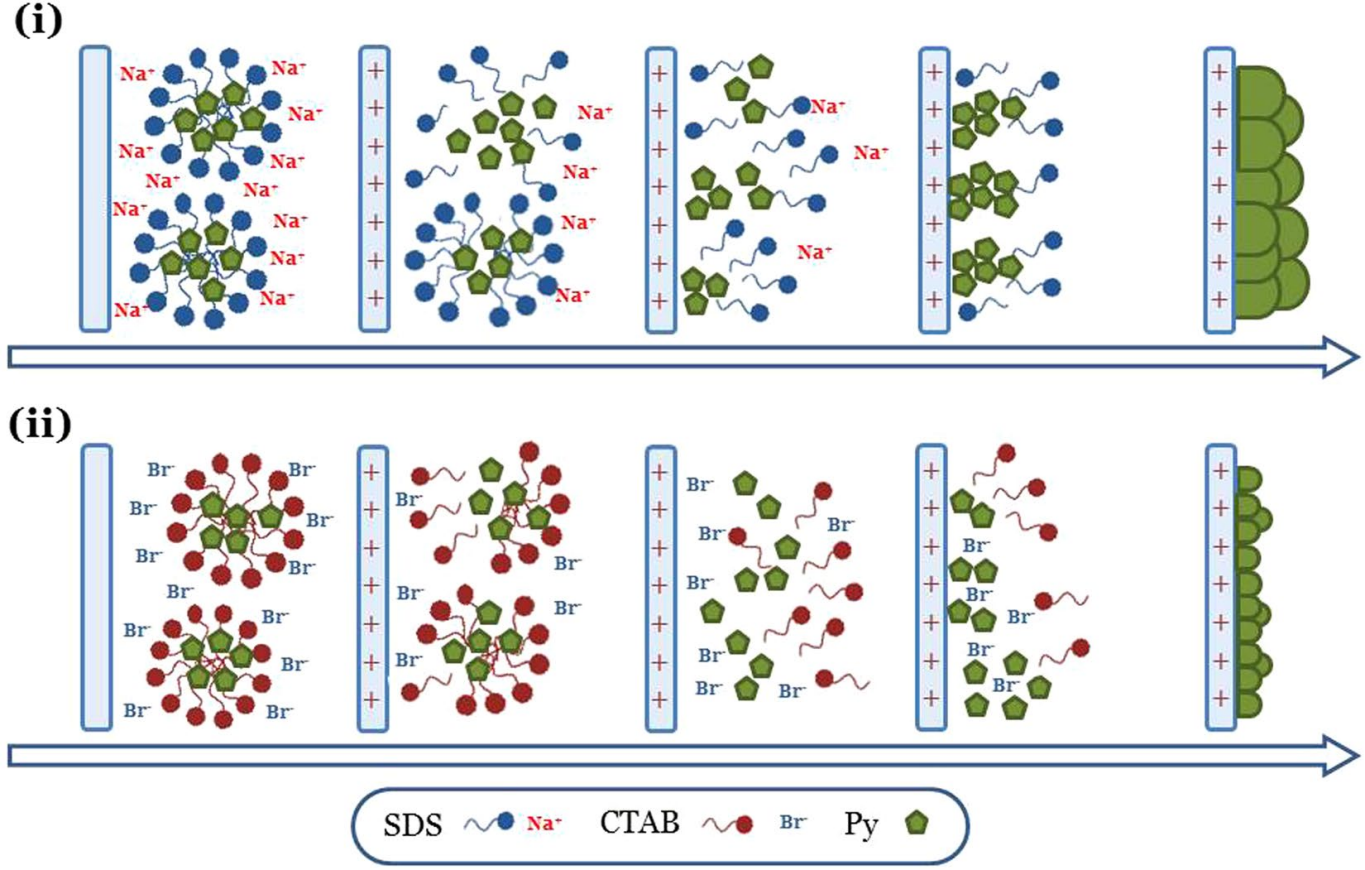
Schematic depiction of the different stages occurring during pyrrole electropolymerization in: (i) SDS solution, (ii) CTAB solution.

As schematized in Fig. 6-i, we can assume that the adsorption process of the anionic SDS surfactant on the positively polarized electrode is favored by the polar heads of the surfactant itself. Such a situation facilitates the approach to the electrode surface of the monomeric units contained in the negative micelles, and hence the subsequent oxidation reaction. In the case of the cationic CTAB surfactant (Fig. 6-ii), the bromide ions are the chemical species affected by the adsorption process across the electrical double layer. Monomers are confined in the positive micelles which constitute an energy barrier to the their interaction with the electrode surface. A valid argument supporting such a mechanism is given by the values of charge consumed during the electropolymerization process (Table 2). In fact it is observed that, for the same polymerization time t = 300 s, the charge consumed for the growth of DSPpy (1050 mC cm^−2^) is nearly one order of magnitude higher than that for CTAPpy (180 mC cm^−2^). This result is in agreement with the fact that the electrochemical yield of the pyrrole polymerization process is higher in SDS than in CTAB solution. However, a polymerization current efficiency of 100% cannot be considered, since also the oxidation of the Ppy backbone is expected to occur during the polymer growth. Therefore it was necessary to perform microbalance measurements in order to obtain the real mass of the deposited polymer (Table 2). It was found that the mass of the polymer from SDS solution (50 μg) is about 5 times higher than that of the polymer from CTAB solution (10 μg). This result further validates the mechanism proposed for the electropolymerization process in the two differently charged surfactant solutions.

All the above considerations can be extended to the synthesis of the composite samples. We can observe an increase of the consumed charge and mass when the GP are used as a filler, with a greater relative rise of the two quantities in the case of using the cationic CTAB surfactant. The mass increase can be reasonably explained by the incorporation of GP into the polymer matrix during polymer growth. However, the increase in the polymerization charges can be attributed both to a higher yield of polymerization, and to a higher level of oxidation in the presence of the graphene filler. A Raman spectroscopy characterization was then performed in order to investigate the molecular structure of the produced polymer systems. In the next section the results of the structural analyses are reported.

#### Structural analysis of PpyGP nanocomposites

**Figure 7:**
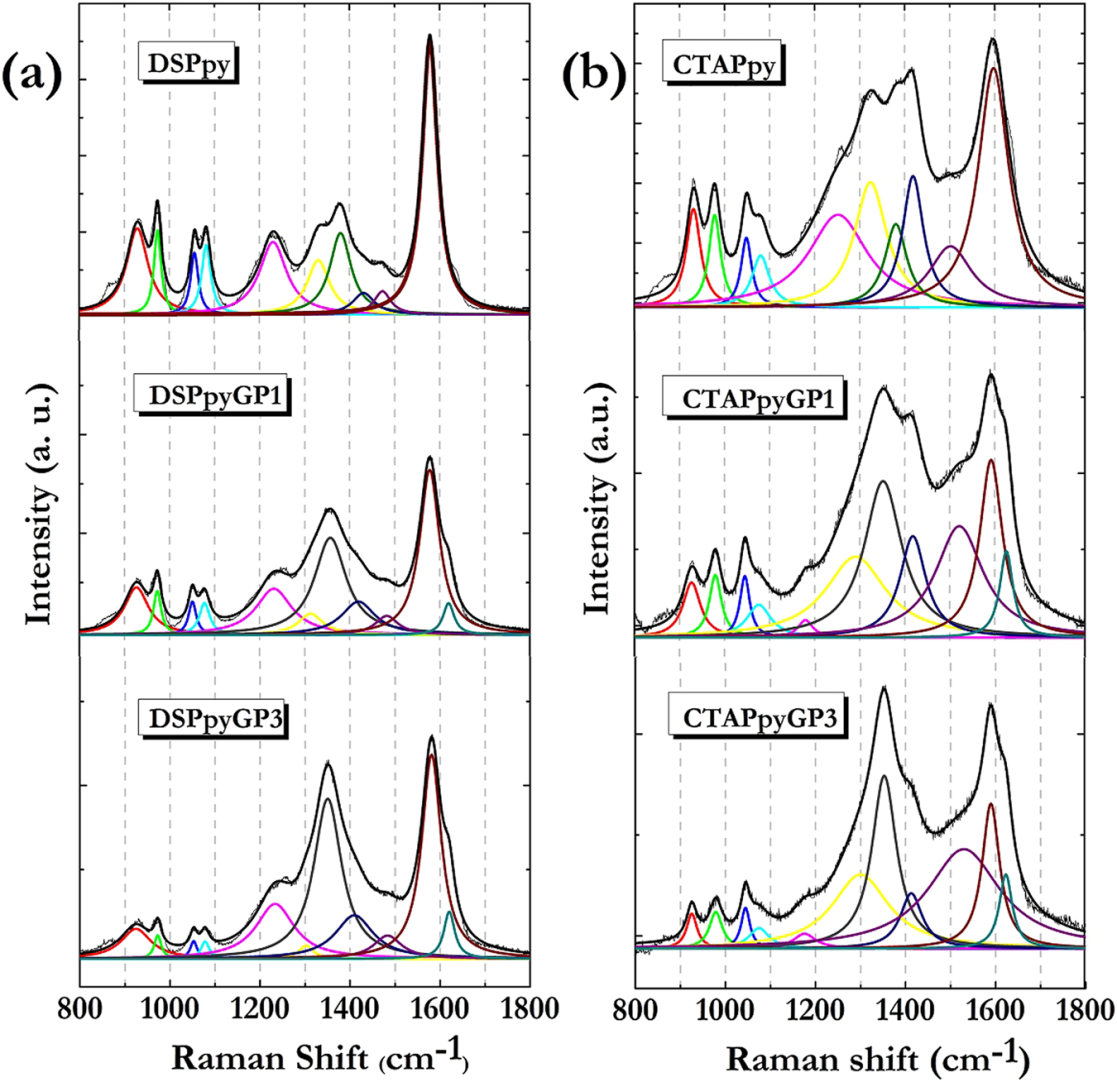
From top to down deconvolved Raman spectra of: (**a**) DSPpy, DSPpyGP1, DSPpyGP3; (**b**) CTAPpy, CTAPpyGP1, CTAPpyGP3.

Figure 7 shows the Raman spectra of all polymeric samples acquired under the 785 nm laser source, along with the curves obtained by the fitting procedure.

The data analysis was carried out by examining the spectral region between 800 and 1800 cm^−1^, that is the frequencies range where the Raman signals related to the polypyrrole main vibration modes are observed. In Table 3 the assignments of the bands deriving from the fitting operation are reported.

**Table 3:** Assignment of Raman bands for polymer and composite samples.

Ppy (cm^−1^)		PpyGP1 (cm^−1^)		PpyGP3 (cm^−1^)		Assignments	Ref.
SDS	**CTAB**	**SDS**	**CTAB**	**SDS**	**CTAB**		
930	931	927	927	926	926	δ_ring_ Ppy^2+^	^88, 89^
974	978	973	980	973	979	δ_ring_ Ppy^·+^	^88, 90^
1056	1049	1051	1045	1051	1045	δ_C-H_ Ppy^·+^	^91^
1082	1081	1078	1076	1078	1075	δ_C-H_ Ppy^2+^	^91^
			1180		1176	δ_C-H_ bipyrrole	^92^
1229	1223	1232		1232		δ ring	^92^
1329	1320	1314	1291	1276	1300	ν_C-C_ inter-ring	^88^
1380	1381					ν_C-C_ inter-ring	^88^
		1357	1352	1353	1353	D band	^49^
1438	1418	1420	1418	1422	1413	ν_C-N_	^93^
1481	1505	1483	1521	1484	1530	skeletal band	^78, 94^
1578	1597	1578	1592	1578	1590	ν_C=C_ Ppy^·+^	^76, 95^
		1620	1626	1620	1623	D’ band	^50, 51^
**I** ***ν***_***C*=*C***_ **/I skeletal band**							
10	4	5	2	7	1		^78^

The signal produced by the C=C symmetric stretching vibration is the feature typically monitored to evaluate the structural changes involving the polymer backbone upon redox processes. The position of such a signal can range from 1550 to 1625 cm^−1^, depending on the oxidation degree of the main chain. Namely, when the vibration signal is located at the lower wavelengths, the polymer is in the totally reduced state (*Ppy*^0^); when the vibration is detected at wavelengths around 1580 cm^−1^, a semi-oxidized state of the polymer is considered ($Pp{y}^{\cdot +}$). Finally, at the higher energies the vibration related to the totally oxidized form (*Ppy*^2+^) is found^76^.

From Table 3 we observe that all spectra related to pure DSPpy and composite DSPpyGP samples show the C=C vibration at a wavelength of 1578 cm^−1^, indicating that the polymer matrix is in the semi-oxidized state. For CTAPpy polymer and CTAPpyGP composite samples, a ∼20 cm^−1^ blue shift of the vibration signal is found, indicative of a slight higher oxidation level of the polymer backbone. Probably, this is due to the higher potential required for the pyrrole polymerization in CTAB solution. Anyway, all the values of the C=C mode wavelengths for DSPpy- and CTAPpy-based samples are broadly within the range of energies associated with the vibration of the semi-oxidized state, that is the one corresponding to the polaron radical cation conducting form of the polymer ($Pp{y}^{\cdot +}$). Therefore, it can be assumed that the incorporation of GP into the polypyrrole matrix has no significant effects on the oxidation state of the polymer. On the other hand, the presence of GP inside polypyrrole is evidenced by the occurrence of the D (~1353 cm^−1^ – grey line) and D′ (~1620 cm^−1^ – dark cyano line) bands in the composites spectra. Conversely, the G band is unclearly identifiable because it is located at the same frequency of the C=C vibration of the polymer, and moreover shows a rather low Raman efficiency under the 785 nm laser excitation source^77^. An additional consideration can be made by comparing the intensity of the C=C stretching and skeletal (∼1500 cm^−1^) bands. In particular the Iν_C=C_/I_skeletal band_ ratio provides a qualitative measurement of the backbone conjugation length, *i.e*. the greater the ratio, the longer the conjugation length^78^. As shown in Table 3, all the samples produced in SDS solution provide values that are higher than those given by the samples grown in CTAB solution.

At this point, a critical reading of overall results allows for visualizing the formation mechanism of nanocomposite materials with a more detail. Firstly, the high conductivity exhibited by the platelets suggests a their direct involvement in the electrodeposition process. In fact, we can suppose that they act as additional nanoelectrodes, able to expand the active surface available for monomer nucleates electro-oxidation. This action is reasonable favored by the oxygenated groups present on GP surface which drive their approach to the positively polarized working electrode during polymerization.

**Figure 8:**
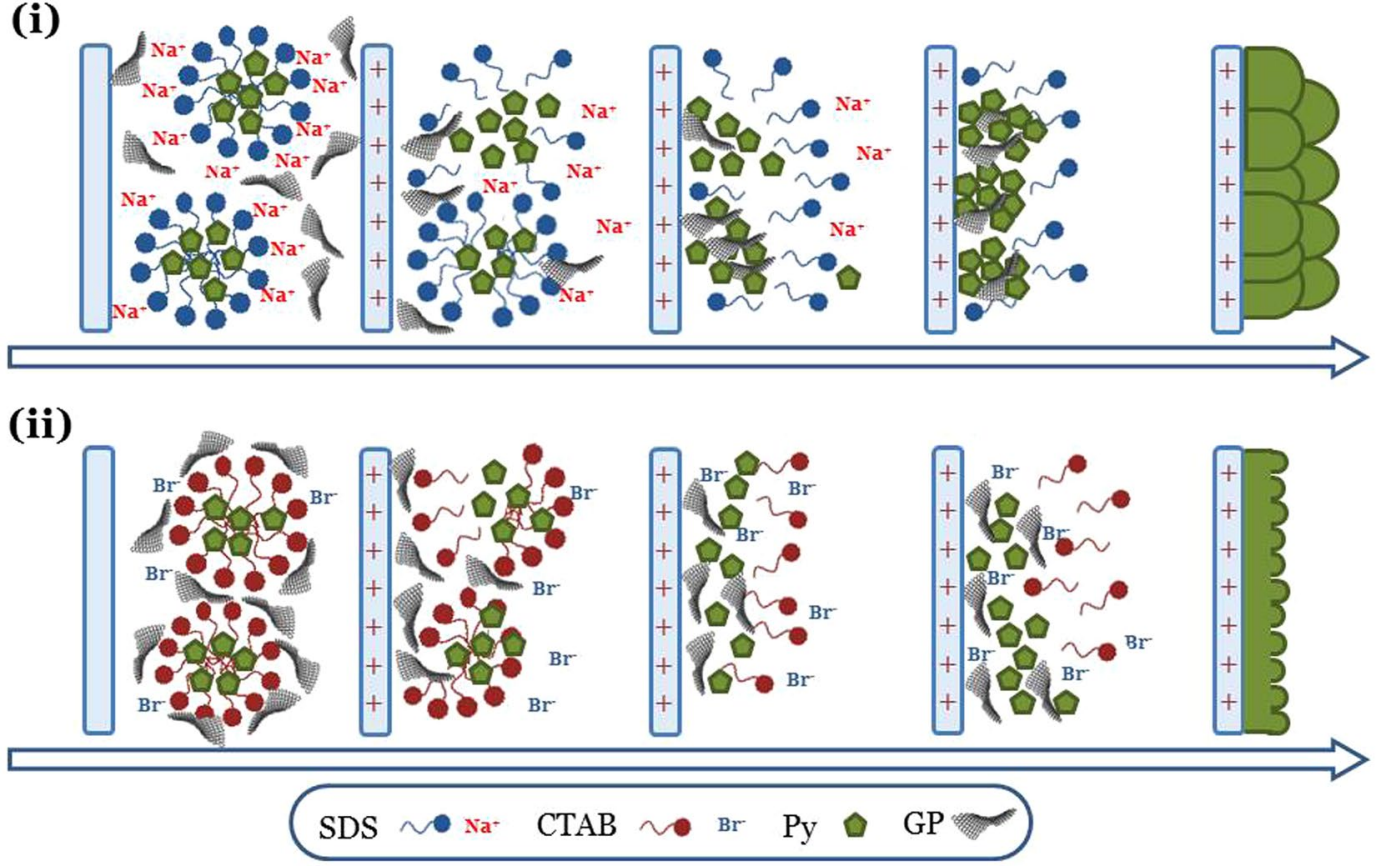
Schematic depiction of the different stages occurring during pyrrole electropolymerization in: (i) GP-SDS dispersion, (ii) GP-CTAB dispersion.

Then, we should consider the probable different interactions rising between the monomer-containing micelles and the nanocarbon phase. In the case of the SDS anionic surfactant, in fact an interaction between the outer cationic coordination sphere surrounding the sulfate polar heads of micelles and the platelets is realistically established (Fig. 8-i). Conversely, in CTAB solution a direct greater affinity between the positive micelles and GP oxygenated surface is expected. In such a situation, the monomer approach to the positive electrode is presumably facilitated (Fig. 8-ii). Indeed this description gets a validation by the syntheses data (Table 2) which point out how the presence of GP during electropolymerization is able to increase up to five times the total mass of the final material (i.e. CTAPpyGP3), against a mere doubling in the case of the DSPpy.

A further support to the proposed formation mechanisms comes from the study of samples morphologies reported in the following paragraph.

#### Morphological characterization of PpyGP nanocomposites

**Figure 9:**
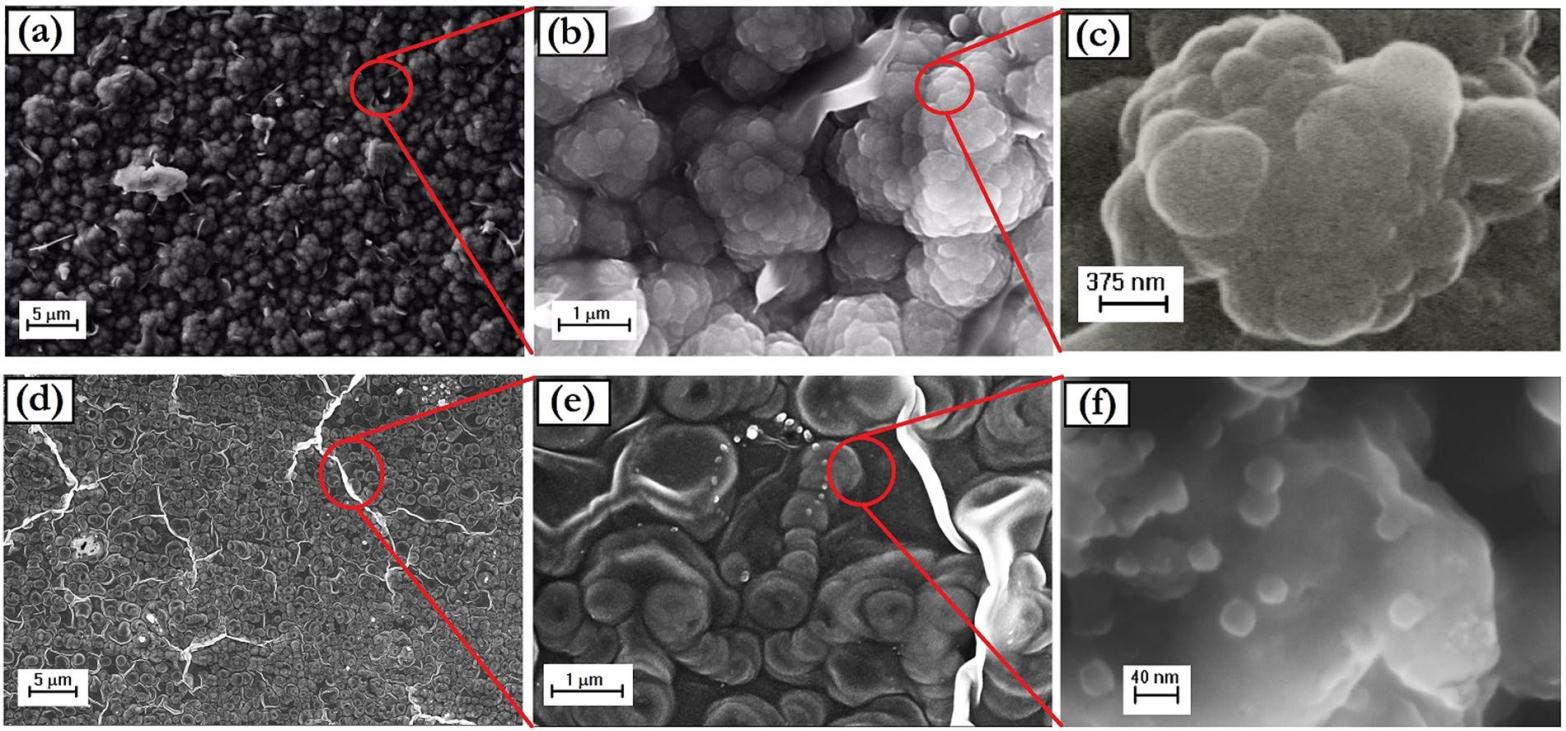
SEM images at different magnifications of: (**a**–**c**) DSPpy; (**d**–**f**) CTAPpy.

The morphology of the pure polymer samples is shown in Fig. 9.

It is evident that the polymer is characterized by specific features dependent on the surfactant employed for the polymerization process. Polypyrrole grown with dodecyl sulfate as dopant (i.e. DSPpy) consists of quasi-spherical units of a few hundred nanometers, aggregated together to form micrometer sized globular entities (Fig. 9a–c). A fine nodular structure, with a nodule mean size of some ten nanometers, is instead observed for polypyrrole electropolymerized in CTAB solution (i.e. CTAPpy) (Fig. 9f). In addition, the macroscopic organization of these last nanostructures appears as a continuous film textured with a multitude of complex protuberances (Fig. 9d,e). A possible explanation of these different morphologies could be that, following the mechanism above discussed and reported in Fig. 6-i, the anionic micelles produced by dodecyl sulfate ions are responsible for the formation of polymer globules by a sort of soft-template effect during the electropolymerization reaction. In the case of CTAB, the cationic micelles produced by cetyltrimethylammonium ions are not able to direct the polymer growth, due to their unfavorable interaction with the working electrode under positive polarization. It is thus conceivable that the polymeric nucleates are arranged in the proximity of the electrode surface outside the micelles structure, giving rise to nanometer nodules compactly assembled to form a film (Figs 6-ii and 9d). In this view, the various lumps protruding from the surface of the CTAPpy sample can be considered as local detachments of the film due to the oxygen evolution at the high overpotential (+1.2 V) required for the pyrrole polymerization.

**Figure 10:**
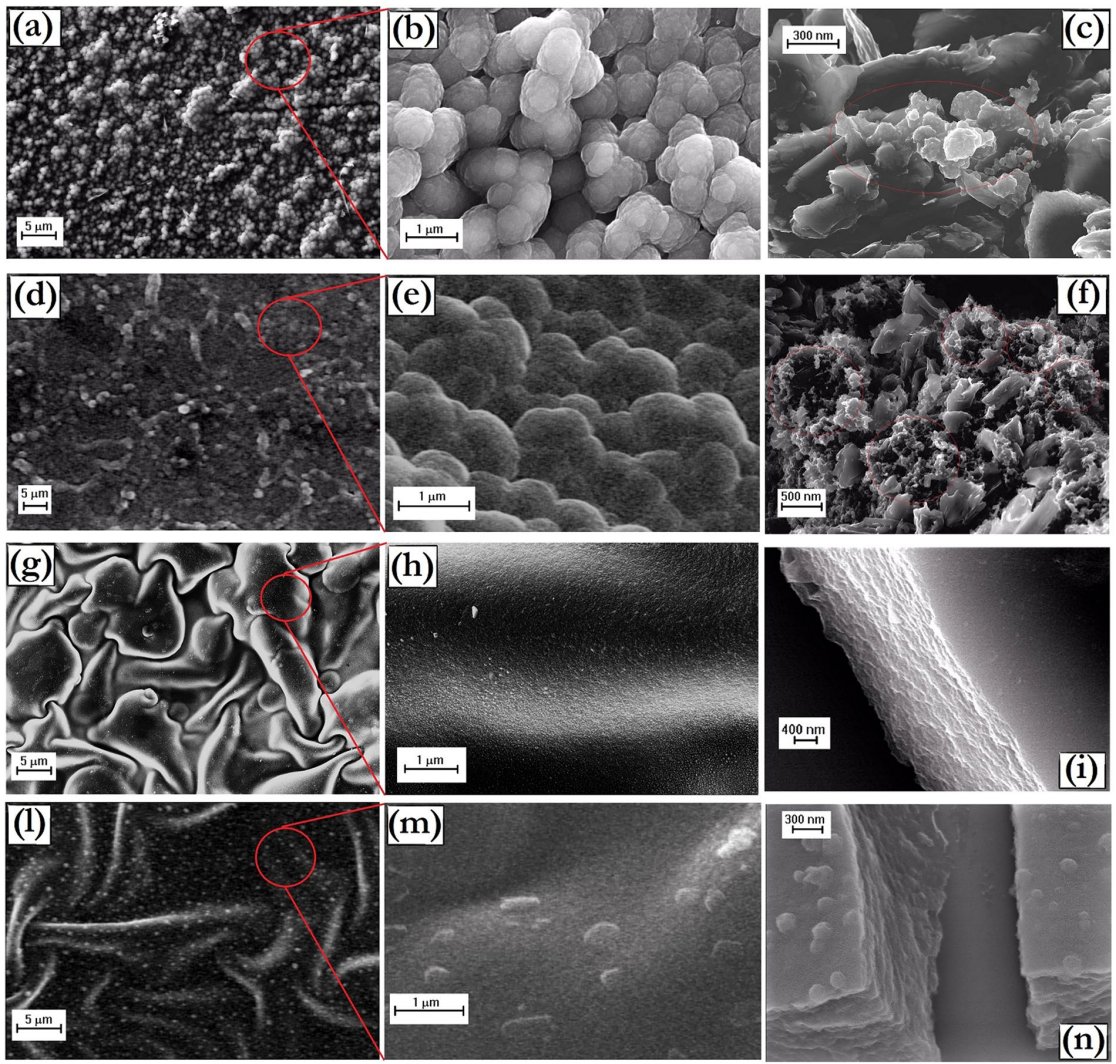
SEM images at different magnifications of: (**a**–**c**) DSPpyGP1; (**d**–**f**) DSPpyGP3; (**g**–**i**) CTAPpyGP1; (**l**–**n**) CTAPpyGP3.

The morphology of the composite films is quite similar to that of the pure polymers. Globular microspheres with a fine nodular nanostructure are found for DSPpyGP1 (Fig. 10a,b) and DSPpyGP3 (Fig. 10d,e) samples. The filler does not alter the template polymerization performed by the SDS surfactant. Moreover, for the highest concentration of filler (DSPpyGP3) a greater compaction of globules seems to occur. Such features are probably induced by the stacking of GP among the globules during the polymer growth (Fig. 8-i). A confirmation to these hypotheses is given by the higher magnification SEM images (Fig. 10c,f) acquired after fracturing the sample surface, which show an evident GP distribution around the smashed polymeric globules (light red circles).

Similarly to pure CTAPpy polymer, in the case of CTAPpyGP1 (Fig. 10g,h) and CTAPpyGP3 (Fig. 10l,m) composites the formation of nanometer roughness films is again found. However, the presence of GP tends to increase the size of the detached film portions. A possible explanation for this phenomenon can be found in the greater affinity of the polymer nucleates for GP than for the electrode surface. In fact, as above discussed, the presence of some oxygen-containing groups on GP probably promotes the adsorption of the positive cetyltrimethylammonium micelles on their surface, thus facilitating the pyrrole polymerization process on their surface as depicted in Fig. 8-ii. The higher magnification SEM images (Fig. 10i,n) taken on fragments of the films support such mechanism, revealing an inner layered structure reasonably produced by the polymer grown on and among the platelets.

#### Electrochemical characterizations of PpyGP nanocomposites

The electrochemical behavior of the pure Ppy and composite PpyGP samples was investigated under the same experimental conditions by recording cyclovoltammograms in 0.1 M tetrabutylammonium perchlorate (TBAP) acetonitrile (CH_3_CN) solution at different scan rates. In order to evaluate the role of the GP filler, the potential window was limited to the range in which can be evidenced the redox reactions related to reversible formation of $Pp{y}^{\cdot +}$ radical cations.

**Figure 11:**
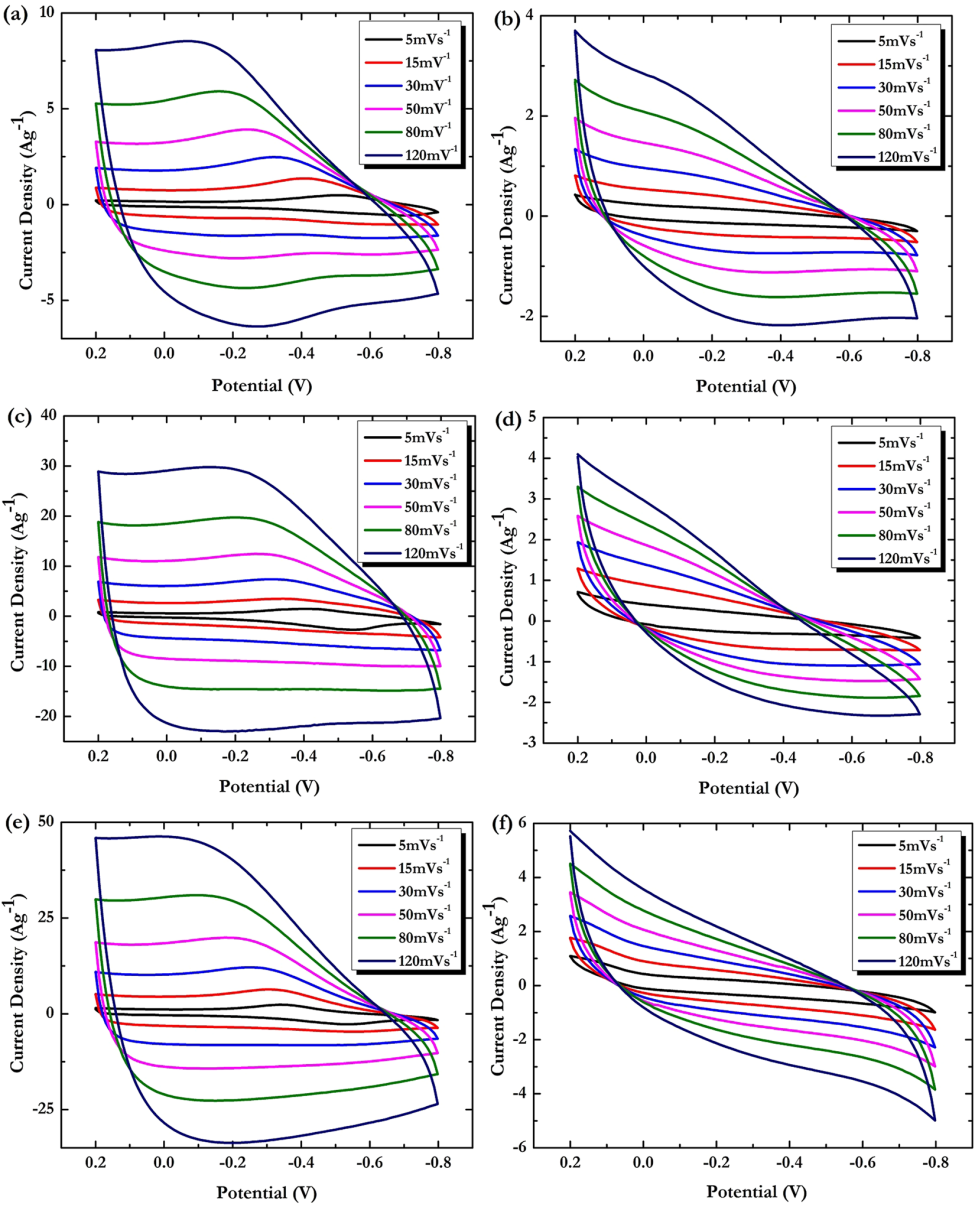
CV characterizations in 0.1 M TBAP in CH_3_CN at potential range 0.2 to −0.8 V vs Ag/AgCl at different scan rates of: (**a**) DSPpy; (**b**) CTAPpy; (**c**) DSPpyGP1; (**d**) CTAPpyGP1; (**e**) DSPpyGP3; (**f**) CTAPpyGP3.

In Fig. 11 are reported the CV taken from the various samples in the potential range from 0.2 to −0.8 V vs Ag/AgCl. Each curve is the average of at least three potential cycles. It is well-known that the shape of CV curves of electroactive polymers are affected by a number of factors including potential cycling range, scan rate, used solvent, electrolyte composition, polymerization conditions, and so on. Moreover, in the course of cycling, the polymer can exchange its initial dopant anion with another one coming from electrolyte. The diffusion processes of these species are typically controlled by the polymer porosity and the nature of the penetrating anion.

From the comparison of the CV curves, it is noted that the presence of GP slightly alters the general feature of the electrochemical response of the polymeric matrices. In fact, even if the peaks positions remain almost the same for the pure polymers and the related nanocomposites, the shape of the voltammograms is substantially changed by the GP filler. Moreover, marked differences are found when considering the current densities recorded during the cycling, with the higher values found for the nanocomposite materials.

The changes involving the polymer matrix during the potential sweeping can follow the two main pathways described by the following redox reactions:

3 $$Pp{y}^{\cdot +}/{A}^{-}+e\rightleftharpoons Pp{y}^{0}+{A}^{-}$$

4 $$Pp{y}^{\cdot +}/{A}^{-}+e\rightleftharpoons Pp{y}^{0}/{A}^{-}/{C}^{+}$$

The predominance of one mechanism on another one depends on the nature of the *A*^−^ dopant counterion. When small mobile anions are used for electropolymerization, the anionic transport predominates and the redox of the polymer backbone will follow eq. (3). On the contrary, when the polymer is synthetized in the presence of bulky immobile anions, the cationic transport through the polymer/electrolyte interface can predominate following eq. (4).

The overall shape of CV for DSPpy, DSPpyGP1, and DSPpyGP3 samples is similar to that found for systems containing large dopant anions for which the cation exchange defined by eq. (4) is the predominant mechanism during the redox reaction^79^. However, a loss of peaks definition associated with a more rectangular line shape is observed for the nanocomposite curves (Fig. 11c,e). At the same time, a significant increase of both anodic/chatodic current densities is recorded when the GP are loaded into the polymer matrix. Such modifications of the voltammetric response are compatible with an electrical double layer (EDL) contribution to the total charge storage process, and it is plausibly due to the GP filler.

In the samples containing the CTAPpy polymeric system, an anion exchange should mainly occur during the redox of the polymer backbone as stated by eq. (3). However, we can observe that redox peaks are poorly detectable in the CV curves of pure polymer (Fig. 11b) up to disappear in those of composite samples (Fig. 11d,f) independently from the scan rate. This electrochemical feature could be related to a mixed diffusion-charge transfer controlled mechanism of the redox reactions of polymer chains. Nevertheless, higher total current density values are recorded for nanocomposites, suggesting that also for these materials an additional EDL capacitance from the GP is likely given.

**Figure 12:**
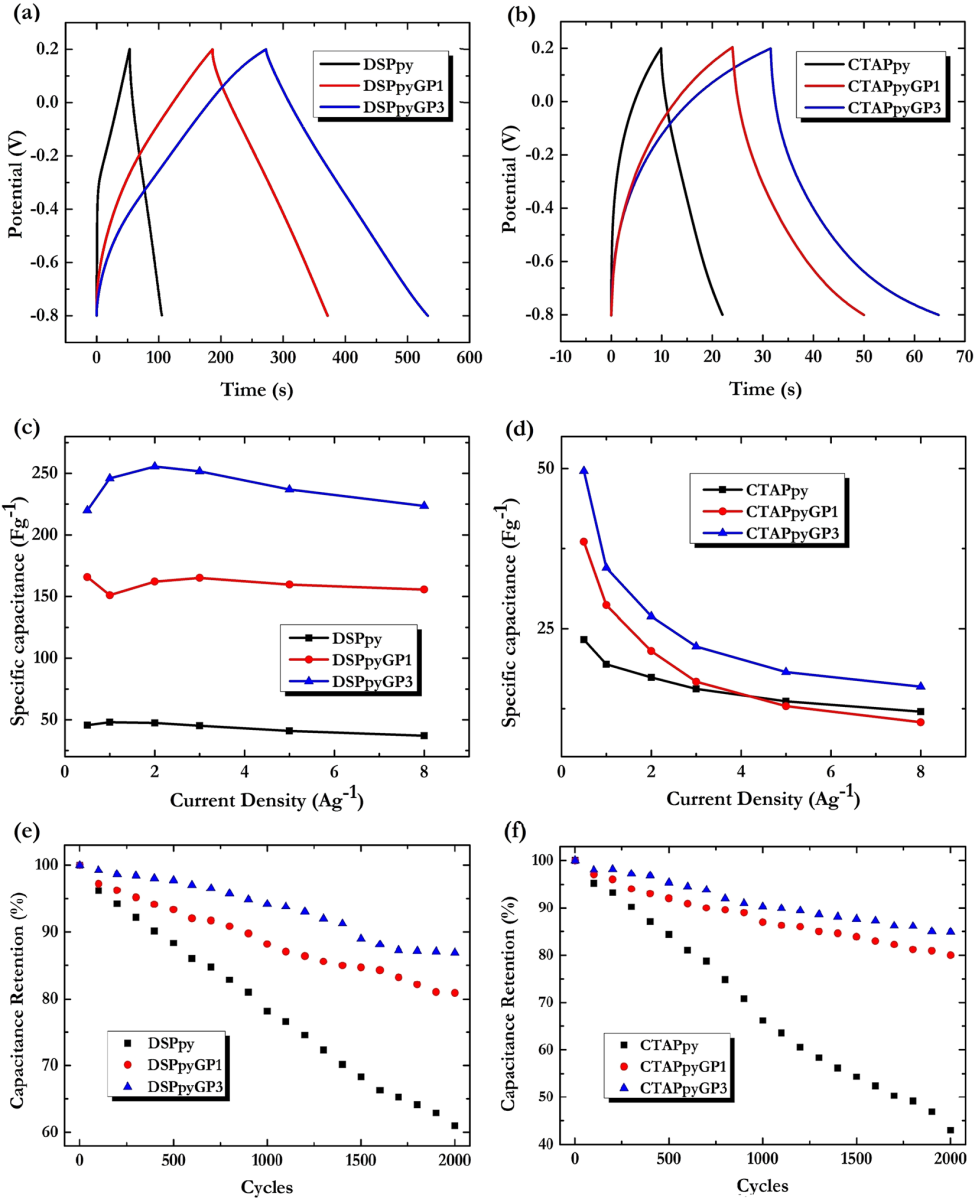
Panel above: GCD curves in 0.1 M TBAP in CH_3_CN at current density of 1 A g^−1^ of: (**a**) DSPpy (black), DSPpyGP1 (red), DSPpyGP3 (blue); (**b**) CTAPpy (black), CTAPpyGP1 (red), CTAPpyGP3 (blue). Panel central: Specific capacitance of: (**c**) DSPpy (black), DSPpyGP1 (red), DSPpyGP3 (blue); (**d**) CTAPpy (black), CTAPpyGP1 (red), CTAPpyGP3 (blue) at different current densities. Panel below: Percentage retention of the specific capacitance as a function of the charge-discharge cycles of: (**e**) DSPpy (black), DSPpyGP1 (red), DSPpyGP3 (blue) at current density of 2 A g^−1^; (**f**) CTAPpy (black), CTAPpyGP1 (red), CTAPpyGP3 (blue) at current density of 0.5 A g^−1^.

In the light of all these outcomes, aiming at better define the capacitive performances of the samples, galvanostatic charge/discharge (GCD) experiments at current densities varying from 0.5 to 8 A g^−1^ were performed in 0.1 M TBAP acetonitrile solution within the potential window −0.8 to 0.2 V (vs Ag/AgCl). In Fig. 12a,b are reported the typical GCD curves of the DSPpy- and CTAPpy-based samples at a current density of 1 A g^−1^.

We can note how the GCD responses agree with the voltammetry results for both sample types. All the nanocomposites in fact exhibit longer charge-discharge times than those of pure polymers, confirming the contribution of GP to the total capacitance of the materials.

In particular, the DSPpy-based systems present quasi-linear variations of potential with time and a negligible *IR* drop, thus by producing GCD curves with a rather symmetrical triangular shape. A different feature is found for CTAPpy-based materials, which instead show an evident deviation from linear variations of the voltage during the charging-discharging process. Such outcomes could depend on the different charge storage mechanisms occurring for the two polymer-dopant matrices as already highlighted by the CV measurements. The higher conjugation lengths found for DSPpy systems probably favor reversible reactions of the polymer chains, and the consequent ions exchange from the polymer bulk to electrolyte during redox processes. For nanocomposites, a decisive role is as well played by the conductive GP, which, dispersed inside the polymer, would increase the electrochemical active interfaces, and at the same time would facilitate electronic hopping among chains during redox reactions by enhancing the capacitive behavior of the hybrid materials.

Quantitatively, an estimation of the specific capacitance *C*_*sp*_ can be achieved by the galvanostatic discharge process according to the equation:

5 $${C}_{sp}=I\times {\Delta }t/(m\times {\Delta }V)$$

where *ΔV* is the voltage change during the discharge process, *m* is the mass (g) of active material for the working electrode, *I* is the applied current density (A), and *dt* is the time of discharge stage^80–82^.

In Fig. 12c,d the specific capacitance variations with current density from 0.5 to 8 A g^−1^ are shown for all the samples. The calculations indicate that the nanocomposites exhibit a higher capacitance than the pure polymers, with the DSPpyGP3 and CTAPpyGP3 reaching about. 250 and 50 F g^−1^ at 2 A g^−1^ and 0.5 A g^−1^, respectively. In addition, the materials synthesized in SDS solution exhibit higher *C*_*sp*_ values. This can be attributed to a better combined effect between the redox and double-layer capacitive behavior of Ppy and GP, respectively. Although each method of material preparation and testing leads to different morphological-structural characteristics and electrochemical performance, it can be seen how a synergistic cooperation with a positive feedback on specific capacitance values has been pointed out for several graphene/polypyrrole systems^83^. For example, graphene/Ppy electrodes prepared by template chemical oxidation polymerizations have shown maximum *C*_*sp*_ values of 160 F g^–1^ at 0.5 A g^–1^ or 457 Fg^−1^ at 0.15 A g^−1^ depending on whether they exhibited a nanofiber^84^ or a nanotubular^85^ morphology, respectively. At the same time, electrochemically deposited graphene/Ppy composites have given specific capacitance of 126 F g^−1^ or 424 F g^−1^ if measured at 100 mV s^−1^ ^86^ or at 1 A g^−1^ ^87^, respectively.

In order to test long time electrochemical performance, the cycle stability was evaluated by repeating the GCD experiments at the current densities for which DSPpy- and CTAPpy-materials exhibited the higher specific capacitance. In Fig. 12e,f we report the percentage of specific capacitance as a function of the cycle number calculated for DSPpy samples at the constant current density of 2 A g^−1^, and for CTAPpy samples at the constant current density of 0.5 A g^−1^, respectively. The capacitance retention of pure polymer keeps 61% after 2000 cycles in the case of DSPpy, and only 43% in the case of CTAPpy. The fact can be attributed to the degradation of Ppy chains induced by the continuous swelling – deswelling during redox processes. On the contrary, the nanocomposites demonstrated superior cycling stability after 2000 cycles with capacitance retention values of 82% and 87% for DSPpyGP1 and DSPpyGP3, and of 79% and 83% for CTAPpyGP1 and CTAPpyGP3, respectively. These results allow us to further underline the synergistic action between the polymer and the nanocarbon phase. Indeed the latter, in addition to increase the interfacial area active for charge transfer and adsorption processes, also proves to be able to improve the mechanical strength and stability of the polymer, preventing its degradation and loss of activity.

## Conclusions

Recent structural analyses performed on the “old” shungite mineraloid evidenced that the building blocks forming the microstructure of its carbon phase can be agglomerates of graphene-like systems. This was the starting point of our research work, because we considered the possibility to extract graphene-based materials from the raw rock, and to assess a novel top-down route for producing surface-controlled graphene nanostructures. In this context, the target of the present work was to settle a methodology for separating graphene platelets from shungite, and to check if the platelets obtained following this manufacturing strategy could be coupled with a polymer to give nanocomposites with specific functionalities. At this aim, the graphene platelets were used for the preparation of polypyrrole-based nanocomposites by using an *in situ* soft-template electrochemical strategy for inserting such remarkable sp^2^-carbon nanostructures inside the polymer matrix.

Specifically, the optimization of the purification protocols allowed us for a 85% yield extraction of thin polycrystalline nanostructured graphite platelets with micrometric in-plane dimensions and an ordered stacking of graphene layers. The GP showed a low oxygen content (5%) and a high electrical conductivity (1490 S cm^−1^). The control of the surface chemistry permitted to modulate the interactions between GP and monomers during the electropolymerization process. Being the latter mediated by the presence of surfactant templates, it was thus possible to discriminate the monomer polymerization mechanisms in the presence of the GP filler and differently charged surfactants.

A series of investigations made us able to describe the nature of the interfaces between GP and the various micelle templates produced by the two different surfactants, and to evidence the mutual interactions between GP and polymer nucleates during the polymerization reactions. This allowed for a rationalization of the morphology exhibited by Ppy polymer in the composite materials produced under dissimilar experimental conditions.

GP were found to increase the surface available for the polymer growth providing a general higher polymerization yield. Moreover, thanks to a synergistic action with the polymer phase, GP demonstrated to favor the transport of both electrons and ions within the bulk material. This was reflected into an enhancement of the charge storage capacity of the nanocomposites with respects the pure polymers. Indeed, the coupling of capacitive (GP) and pseudo-capacitive (Ppy) storage mechanisms acting into nanocomposites enabled to achieve specific capacitance values up to 250 F g^−1^ at a 2 A g^−1^ discharge current density.

In conclusion, on the basis of their chemical and physical features, it was assessed that the 2-D graphene-like nanomaterials obtained from SH source are suitable active filler for Ppy-based nanocomposites that can find application in a very broad range of technologies. In fact, considering the biocompatible of both graphene and Ppy, such nanocompisites could indeed be successfully exploited also for bio-related applications like artificial muscles, neural interfaces and so on.

The rationalization of the processes providing polymer composites with proper morphology and structure, and characterized by defined functionalities lays the basis for the manufacturing of versatile systems with customizable electrochemical performance.
